# Supplementary material for: Spatial Control of Light‐Responsive Proteins and Optogenetics Within Hydrogels via Volumetric Bioprinting
Source: Adv Mater. 2026 Aug 10;38(60):e74563. doi: 10.1002/adma.74563 (PMC13712202; doi:10.1002/adma.74563)
Supplement: Supplementary file 1 [file ADMA-38-e74563-s002.docx]

**Supplementary Information for:**

**Spatial control of light-responsive proteins and optogenetics within hydrogels via volumetric bioprinting**

*Davide Ribezzi, Pere Català, Gabriel Größbacher^1,2^, Paulina Nuñez Bernal^1,2,3^, Olaf Nijssen^1,2^, Sammy Florczak^1,3^, , Wilco Nijenhuis^4,5^, Nuria Crusellas-Villorbina^1,3^, Bram Nijhoff, Paul Delrot, Jos Malda, Andreas Hierholzer, Martin Fussenegger, Lukas C. Kapitein, Riccardo Levato^,*^*

*Correspondence to : [r.levato@uu.nl](mailto:r.levato@uu.nl)

**SUPPORTING INFORMATION**

**Supplementary methods**

***Optical density measurements for GelMA and AlgMA***

GelMA (5% w/v) and AlgMA (3% w/v) solutions were prepared by dissolving the pre-polymers in PBS, and supplemented with 0.1% w/v LAP, and either mixed with HeLa cells (2 million/mL) or left cell-free. Cell-laden and cell-free suspensions were placed in a 96-well multiwell plates (250 uL/sample), and photocrosslinked (405nm, 3.1 mW/cm2, 5 minutes). Pre-crosslinked, cell-free solutions were also added, together with a PBS-only control. All groups were included with 3-4 replicates. Optical density at 405nm was measured with a spectrophotometer (CLARIOstar Plus, BMG Labtec), and absorbance values were recorded.

**Alginate photoactivation assays**

Alginate methacrylate was dissolved in PBS to 3% (w/v) and mixed with HeLa mEos3.2 cells at 2 × 10⁶ cells/mL. The resulting bioink was cast under a 405 nm lamp for 2 minutes (delivering a light dose of 372 mJ/cm^2^) and transferred to a cuvette. A dose test was then performed on a volumetric printer (Tomolite v2, Readily3D, Switzerland) using its Apparite software, by projecting a matrix of circular spots each illuminated at a different light dose (1000, 2500, 5000, 7500, 14000, and 20000 mJ/cm²) obtained with variable light intensity (20, 30, 40, and 50 mW/cm²) (n = 3).

***Characterization of the support bath for Embedded extrusion Volumetric Printing***

To perform as a suitable bath for embedded extrusion printing, the low molecular weight GelMA needs to allow for the movement of the immersed needle, while offering structural support to the extruded filament and while preserving its stress-recovery capacity. To characterize and confirm this behaviour, a shear strain sweep test was initially performed, and results showed a cross point of *G*′ and G″ at 440% strain, resulting in a solid-liquid transition (**Figure 3B**, in the main body of the manuscript). Secondly, a shear rate ramp was performed and a shear thinning behaviour was observed as the shear stress increased in a nonlinear fashion and, under the same conditions, viscosity decreased (**Figure 3C**). Finally, stress/recovery tests were performed by alternating low (1%) and high (500%) strain at 1 Hz frequency. Results showed how, when the higher strain was applied, higher G″ values were measured compared to the *G*′ ones, (**Figure 3D**), proving how the fluid bulk support bath transitioned from a solid to a liquid-like state when a strain was applied, representing the needle translation through the bath during the extrusion phase. Moreover, higher *G*′ values were registered compared to *G*″ after the strain removal, showing an elastic recovery, thus proving the ability of support for the extruded filament. Following the printability optimization, filaments of width ranging from 331 ± 23 µm to 134 ± 22 µm were extruded with a methylcellulose-based ink, previously designed and characterized.^[6]^ With the range of width values achieved, a filament smaller or bigger than the diameter of the needle (27G) utilized in this study could be achieved (**Figure 3E**).

***Cell viability of primary stem cells under photoconversion light dose regimes***

Mesenchymal stromal cells (MSCs) were isolated from equine bone marrow as previously described, [SR1] and cultured in αMEM (Gibco) supplemented with 0.2 mM L-ascorbic acid 2-phosphate (Sigma), 10% FCS (Lonza), 100 U/mL penicillin with 100 µg/mL streptomycin (Life Technologies) and 1 ng/mL bFGF and used at passage 5.  All animal tissue and cells used in this study were obtained from deceased equine donors, donated to science by their owner, and according to the guidelines of the Institutional Animal Ethical Committee. 2x10^6^ MSCs were volumetrically printed in 5% GelMA into cylindrical samples (2mm height, 6 mm diameter) at a light dose of 180 mJ/cm^2^. Samples were kept in culture overnight to wash out any excess photoinitiator and subsequently exposed to additional light irradiation to mimic the highest photoactivation regimes used on the HeLa mEos3.2 experiments (11.25 and 11.73 J/cm^2^, for static and tomographic 3D photoactivation, respectively). Irradiated samples, and non-irradiated controls (n = 3) were cultured for 3 days. Live/Dead assays were performed according to manufacturer’s protocols (ThermoFisher) at days 1 and 3 to assess cell viability post-irradiation. Samples were imaged using a Thunder microscope (Leica) and percentage of live cells calculated using FIJI.

***Assessment of the temporal expression of PDX1 and MAFA after NIR illumination***

HeLa cells were plated in 96-well plates at a cell density of 10000 cells/well. The day after plating, the medium was refreshed, and HeLa cells were transfected with both plasmid constructs as previously described. 24 h post transfection, 2D transfected HeLa cells were exposed to pulsed light regimes of 30 s ON/180 s OFF using 740 nm LED (Roithner, LED740-01AU), 25 mW/cm^2^, connected to an Arduino Mega (microcontroller board, Arduino) via a LED DRIVER (Infineon ILD6150), in phenol red-free cell culture media supplemented with 25 µM biliverdin (30891, Sigma-Aldrich) overnight. Cells were analyzed at different timepoints after induction of light exposure via immunofluorescence staining as described below. Following optogenetic activation 2D-cultured cells were fixed in 4% formaldehyde solution for 15 min at room temperature and permeabilized with 0.1% (v/v) Triton X-100 in phosphate buffered saline for 10 min. After permeabilization, cells were blocked with a blocking buffer (1% (w/v) BSA solution in PBS) for 1 h at room temperature. The cells were incubated overnight at 4°C with primary antibodies goat polyclonal anti-Pdx1 (1:300 dilution, R&D systems) and rabbit monoclonal antiMafA [D2Z6N] (1:800, Cell Signaling Technology) diluted in blocking buffer. After primary antibody incubation the cells were washed three times with blocking buffer and then incubated with secondary antibodies donkey anti-goat AlexaFluor 488 (1:300 dilution, Thermo Fisher Scientific), donkey anti-rabbit AlexaFluor 555 (1:300 dilution, Thermo Fisher Scientific), and 1 μg/mL Hoechst 33342 (Thermo Fisher Scientific) diluted in blocking buffer for 1 h at room temperature in the dark. Samples were then washed three times in PBS and analyzed on a Leica Thunder inverted microscope. Signal quantification was performed in Fiji (ImageJ) by threshold-based segmentation of Hoechst-stained nuclei followed by watershed separation to determine nuclei counts per image.

***Viability of HeLa cells after transfection and encapsulation***

HeLa cells were plated in 6-well plates at a cell density of 800000 cells/well. The day after plating, the medium was refreshed, and HeLa cells were transfected with both plasmid constructs as previously described. 24h post transfection, cells were encapsulated into 6 x 2 mm GelMa discs at a cell density of 2e6 cells/mL. Cell viability was assessed 24h and 72h post encapsulation by staining the constructs with 2 µM Calcein AM and 4 µM ethidium homodimer-1 (EH) for 30 min at room temperature in the dark, and imaged on a Leica Thunder microscope. Live-dead cell quantification was performed in Fiji (ImageJ). Images were treated with a sharpen filter to facilitate by threshold-based segmentation applied independently to the Calcein AM and EH channels, followed by watershed separation to identify and count individual cells. The numbers of live (Calcein AM-positive) and dead (EH-positive) cells were determined for each image, and cell viability was calculated as the percentage of live cells relative to the total number of cells (live + dead).

**Supplementary results**

***Photostimulation of HeLa-mEos3.2 embedded in AlgMA***

As a proof-of-concept demonstration of applying our approach to different classes of biomaterials, we tested Alginate Methacryloyl (AlgMA) as a polysaccharide-based bioresin.(Supplementary Figures S3, S4, and S5). Also in AlgMA, we observed how the dose needed for printing (approximately 372 mJ/cm^2^ ) is considerably lower (approximately 3-fold) than the minimum dose required to activate the mEos3.2 protein, and that the efficiency of photoconversion increases with the applied dose. For the AlgMA-based bioresin, a spatial activation accuracy of 84 ± 3%, with an efficiency of 72 ± 4% of cells converted were measured. As additional characterization, we assessed also the optical density of the AlgMA-bioresin at 405nm, which increased approximately 3-fold over cell-free hydrogels when cells were embedded. This is in line with the increased opacity of light-scattering, cell-laden structures. Although the overall optical density of crosslinked AlgMA 3% was found to be significantly lower (p<0.05, t-test, n=3) than what we measured for our GelMA 5%-based formulations, a direct comparison should be considered with caution, as the two materials are vastly different in composition, polymer content, molecular weight and degree of functionalization. Overall, our data underlines that photoactivation is also possible with this alginate-based material.

**Supplementary figures**

**Supplementary Figure S1**: A) Representative fluorescence images of photoconverted HeLa mEos3.2 (magenta) exposed to the complete grid of screened dose and light intensity conditions for the characterization of the dose dependency behaviour of mEos3.2 photoconversion in 2D. Scale bar: 500 µm. B) Plotted percentage of photoconverted cells in each of the tested conditions (n = 3) and results of the two-way ANOVA showing that dose is the only significant factor affecting photoconversion efficiency.

**Supplementary Figure S2**: A) Fluorescence images and B) percentage of native (blue) and photoconverted (magenta) HeLa mEos3.2 cells pre-treated with doxycycline and embedded in 5% GelMA i) before crosslinking (not irradiated), ii) crosslinked in the volumetric bioprinter at a light dose of 180 mJ/cm2, and iii) fully converted at a dose of 10.2 J/cm2 using the 405 nm laser of a Thunder microscope (n = 5). Scale bar: 200 µm. Data represent mean ± SD. Significance was determined by one-way ANOVA: **** = p< 0.0001.

**Supplementary Figure S3**: Fluorescence images of native (blue) and photoconverted (magenta) HeLa mEos3.2 cells pre-treated with doxycycline and embedded in 3% AlgMA via photocrosslinking at a dose of 372 mW/cm^2^, demonstrating that the light dose required to crosslink the cell-laden material does not lead to photoconversion. Scale bar: 200 µm.

**Supplementary Figure S4**: A) Screening of volumetric printing set-up light intensity and total light dose required for HeLa-mEos3.2 photoconversion in 3% AlgMA samples (n = 3). B) Optical density at 405nm for AlgMA solutions, cell-free and cell-laden crosslinked hydrogels. Data is represented as mean ± SD. Significance was determined by one-way ANOVA: **** = p< 0.0001.

**Supplementary Figure S5**: A) Photoconversion accuracy in AlgMA. Scale bar: 500 µm.

**Supplementary Figure S6**: Optical density at 405m for GelMA solutions, cell-free and cell-laden crosslinked hydrogels. Data is represented as mean ± SD. Significance was determined by one-way ANOVA: **** = p< 0.0001.

**Supplementary Figure S7**: 3D photoconversion of HeLa mEos3.2 in a bioprinted lymph node-shaped model (volume = 190 mm^3^). i) STL model showing the printed, perfusable lymph structure (beige/yellow) and the desired photoactivated regions (magenta). ii) Manual perfusion of the volumetrically printed channels with an alcian blue dye. iii) Fluorescence imaging tilescan of the whole construct showing the photoconverted regions. Scale bar: 3 mm.

**Supplementary Figure S8**: Cell viability at day 1 and 3 of primary mesenchymal stromal cells from equine bone marrow printed within 5% GelMA (180mJ/cm2 dose) and subsequently irradiated at a low dose, and a dose just above the highest photoactivation regime used on the HeLa mEos3.2 experiments (11.73 J/cm^2^) using a volumetric bioprinter (n = 3). Data represent mean ± SD. No significant differences were determined by two-way ANOVA.

**Supplementary Figure S9**: GRACE workflow schematics. Briefly, a light sheet rapidly scans and maps the printing volume in 3D to extract positional, morphometric, and spectral (i.e., fluorescence) information from the contents of the printing vial. This serves as input for multi-parametric modelling algorithms to generate precisely a targeted geometry, which is finally converted into the STL model to be volumetrically printed. Scale bar for panel iii: 500 µm.

**Supplementary Figure S10**: GRACE obstacle correction schematic explanation for the selective photoconversion of HeLa-mESO3.2.

**Supplementary Figure S11**: Live/Dead essay on encapsulated HeLa cells over a period of 7 days.

**Supplementary Figure S12**: Recovery of baseline protein expression over time after photostimulation of the NIR-sensitive circuit, for A) PDX1 and B) MAFA, normalized to the average of the expression level found 24h after induction of photostimulation (n=6, * p = 0.021, **** p <0.0001, one-way ANOVA).

**Supplementary Figure S13**: Viability of HeLa cells in their native state or after transfection with the NIR-sensitive circuit, after embedding in GelMA. Transfected cells display lower viability compared to non-transfected, albeit the overall percentage of living cells is ⁓70% (n=4-5, ** p = 0.042, *** p = 0.0008, two-way ANOVA).

**Supplementary Figure S14.** NIR optogenetic system displays intensity-dependent activation in 3D cultures. (A) A custom illuminator device was built to illuminate the wells of a 96-well plate with programmable light intensity. A microcontroller controlled a custom illumination script consisting of 30 s ON and 180 s OFF cycles, with the average intensity independently adjustable for each row of wells. To shield samples from ambient light, the plate was enclosed in a black 3D-printed box. In each well, a 4 × 1 mm GelMA disks was placed, submerged in medium, and illuminated according to the script for 24 h. (B) 5× IF stainings of Pdx1/MafA expression in GelMA disks after illumination at increasing light intensities. Top values represent % of total LED power, whereas bottom values are absolute intensities. Nuclei are visualized with HOECHST. S.B. = 500 μm. (C) Quantification of Pdx1/MafA expression based on average (avr) fluorescence by cell nuclei count in GelMA disks after illumination at increasing light intensities. Y-axis displays average image fluorescence per number of nuclei, normalized to the unilluminated control (N = 1, n = 3). Data represent mean ± SD.

**Supplementary Figure S15.** Scattering profiles of direct NIR light projections in volumetrically printed cell-laden GelMA constructs. (A) Schematic of the projection setup used for measuring scattering profiles. i: The printing vial was submerged in demineralized water inside a cuvette. The volumetrically printed construct was positioned in phenol red–free medium supplemented with 25 μM BV. A 0.532 mm × 4 mm laser beam was projected through the cuvette onto the target region within the printing vial. ii: Zoom view of an example cell-laden cylindrical construct, standing on a base, submerged with the medium, including a hypothetical scattered light profile. iii: Schematic of Mie scattering of the light by the cells in the print. (B) i: Contrast-reduced preview from the IR-camera side perspective showing projections passing through the construct. (ii) Schematic of the measured profiles: longitudinal intensity (shown in panel D) and perpendicular light diffusion (shown in panel E). (C) Raw images of projections passing through different constructs, rotated 90° counterclockwise. S.B. = 1 mm (D) i: Beam attenuation measured by pixel intensity normalized to start at 0 at x = 0.7 mm to eliminate edge artefacts. Exponential decay curves were fitted to condition average profiles (n = 2). ii: Average percent light attenuation per mm travelled through the construct, derived from decay fit curves. (E) Perpendicular intensity profiles measured by normalized pixel intensity. Grey dotted line at y = 0.532 represents the edge of the projection. ii and iii are zoom-ins of i. ‘▲’ indicates laser input location. Avr. = Average, o.p. = overpolymerized, Pix. = pixel, PRF = phenol-red free.

**Supplementary Figure S16.** Pilot experiments of spatially patterned optogenetic stimulation using NIR-light projections from the bioprinter. Projection of a star pattern (arm width 0.5 mm, axis length 2.4 mm) into bioprinted constructs enables patterning of gene expression for (i) Pdx1 and MafA with low signal in off-target areas, and higher signal on-target. Although off-target activation of cells outside the projected pattern was also observed, particularly close to the border of the hydrogels, were scattering is accentuated by the transition from the culture media to the GelMA, (ii) zonally confined optogenetic stimulation successfully resulted in a 6.28 ± 2.58 (Pdx1) and 5.12 ± 0.79 (MafA) fold increase in the density of activated cells within the star pattern. The overall density of higher signal cells in the on-target areas is approximately 5-fold compared to the off-target region (N = 2). Scale bar in F is 1 mm, inset is 500 µm.

**Supplementary Video SV1**. Spatial selective activation and photoconversion of mEos3.2-carrying cells.

**Supplementary File 1.** High resolution image for the fluorescence image in Figure 2, Panel B.

**Supplementary File 2.** High resolution image for the fluorescence image in Figure 2, Panel C.

**Supplementary File 3.** High resolution image for the fluorescence image in Figure 2, Panel E.

**Supplementary File 4.** High resolution image for the fluorescence image in Figure 2, Panel I.

**Supplementary File 5.** High resolution image for the fluorescence image in Figure 2, Panel J.

**Supplementary File 6.** High resolution image for the fluorescence image in Figure 3, Panel Fi-iii.

**Supplementary File 7.** High resolution image for the fluorescence image in Figure 3, Panel Fiv.

**Supplementary File 8.** High resolution image for the fluorescence image in Figure 3, Panel G.

**Supplementary File 9.** High resolution image for the fluorescence image in Figure 3, Panel H.

**Supplementary File 10.** High resolution image for the fluorescence image in Figure 4, Panel H.

**Supplementary File 11.** High resolution image for the fluorescence image in Figure 4, Panel K.

**Supplementary File 12.** High resolution image for the fluorescence image in Figure 4, Panel L.

**Supplementary File 13.** High resolution image for the fluorescence image in Figure 6, Panel C.

**Supplementary References**

[SR1]	R. Levato, Webb W. R., Otto I. A., Mensinga A., Zhang Y., van Rijen M., van Weeren R., Khan I. M., Malda J.. *Acta Biomater*. **2017**, *61*, 41-53.
